# Spatial data and workflow automation for understanding densification patterns and transport energy networks in urban areas: The cases of Bergen, Norway, and Zürich, Switzerland

**DOI:** 10.1016/j.dib.2022.108290

**Published:** 2022-05-18

**Authors:** Remco Elric de Koning, Rogardt Heldal, Wendy Tan

**Affiliations:** a Western Norway University of Applied Sciences, Inndalsveien 28, Bergen 5063, Norway; b Wageningen University and Research, P.O. Box 9101 HB, Wageningen 6700, the Netherlands

**Keywords:** Spatial analysis, Space syntax, Geographical information systems, Urban structure and form, Transport energy usage

## Abstract

A better understanding of how the spatial configuration of cities, understood as urban structure and forms, can achieve sustainable development is needed. This paper presents spatial data and an automated workflow for studying the urban structures (i.e., road and transportation networks) and forms (i.e., building size, position, function and density) of two medium-sized European cities - Bergen, Norway and Zürich, Switzerland. The data focuses on examining correlations between the densification patterns and transport energy usage of these cities de Koning et al., (2020). Spatial and tabular datasets for (i) urban structures, (ii) urban forms, (iii) building density, (iv) road centre lines and (v) transport energy usage are obtained as georeferenced files from OpenStreetMap (OSM) and upon request from collaborating local and national authorities. Transport energy data is derived from traffic data collected from the Norwegian Public Road Authorities or simulated via a traffic model. Open-source data is used wherever possible. Data gaps within proprietary data are supplemented with proxies or open-source data.

Hand-drawn axial maps drawn by the authors using the Space Syntax methods and analysed via depthmapX software are a crucial dataset presented here. All analysed data are then returned to a Geographical Information System (GIS) platform and processed via an automated workflow of 19 steps built via the ModelBuilder^TM^ tool in ESRI® ArcGIS. The automated workflow allows for repetitive cross-city comparison and the compilation of diverse spatial data sources for analysis.

In combination with the novel workflow, the dataset can be used for future comparative studies in spatial planning, transport planning and management of energy systems to facilitate informed decision-making towards more sustainable developments.

## Specifications Table

**Table:** 

Subject	Social Sciences
Specific subject area	Planning and Development
Type of data	Spatial data files (ESRI shapefile) and supporting tables VBA script code ArcGIS Toolbox
How the data were acquired	Part of the spatial data files are georeferenced axial maps hand-drawn by researchers and validated with local experts. The rest were secondary data downloaded from open-source online resources via ArcGIS Editor for OpenStreetMap (OSM). Information on transport capacity and building use were proprietary data provided on request by local authorities (Bergen Kommune) and national authorities (the Norwegian Public Road Administration) and the Institute for Transport Planning and Systems at ETH Zürich as georeferenced shapefiles.
Data format	Shapefile (.SHP), Code Page file (.CPG), dBase Database file (.DBF), Shape Index file (.SHX), Projection file (.PRJ), Extensible Markup Language file (.XML),
Description of data collection	Axial maps were drawn over the street networks of Bergen, Norway and Zürich, Switzerland using the Space Syntax method in ArcGIS. They are exported per city, analysed with depthmapX and then re-imported to GIS. Open-source data for all available street networks, buildings, and plots were downloaded for both cities' geographical and political boundaries via OSM. In addition, proprietary data were obtained on request for both cities.
Data source location	• Bergen, Norway: Latitude: 60.339371 – 60.426075 Longitude: 5.266552 – 5.381914 • Zürich, Switzerland: Latitude: 47.351269 – 47.402858 Longitude: 8.489675 – 8.578846
Data accessibility	The data is hosted at GitHub. Repository name: HVL_PhD, HVL_PCS953 Spatial data: https://github.com/redekoning/HVL_PhD Geo-processing toolkit: https://github.com/redekoning/HVL_PCS953
Related research article	de Koning, R. E., Tan, W. G. Z. & van Nes, A., Assessing Spatial Configurations and Transport Energy Usage for Planning Sustainable Communities. Sustainability, Vol. 12:19 (2020), 8146. 10.3390/su12198146

## Value of the Data

• Comparable data on densification patterns and transport energy from cities in different countries are not readily available. Data on urban form, building density and road centre lines are found at varying units and levels of scales (i.e., neighbourhood and citywide). However, the comparison is essential for drawing insights on spatial relationships to achieve sustainable development.
• The dataset contains spatial data such as building, plots and street networks attached with non-spatial data of building functions and simulated transport energy usage. These georeferenced shapefiles are usable across multiple GIS platforms and allow other researchers to compare with their own cities.
• The primary data of hand-drawn axial maps of Bergen and Zürich are georeferenced and validated. Furthermore, this unique empirical data is confirmed with local authorities and experts involved in the research project available to other researchers.
• The data for urban structure carries the metrics of 'betweenness' and 'closeness' calculated based on Space Syntax theories and methods [2,3] via depthmapX. These metrics improve current geographical and transport planning approaches of proximity or access through speed and distance to improve planning decisions for sustainable mobility.
• The data for urban form (dimensions, age and functions) and building density (function, floor space and plot sizes) are calculated with the Mixed-Use Index (MXI) calculator designed for this workflow. Decision-makers of local, regional and national authorities for spatial planning, transport and infrastructure planning, and resource planning can use this to consider how space is distributed, if the distribution is efficient, and if the content of the distribution can facilitate liveability and sustainability.
• The unique data on workflow automation allows for repetition of data input by other researchers using other cities while allowing for generalisability across different cases. The knowledge and insights can be helpful for strategic spatial and transport planners, spatial development policymakers and road engineers to make well-informed decisions on how to develop urban areas more sustainably and offer methods for analysing cities.

## 1 Data Description

This article provides data on urban structures and forms from Bergen, Norway and Zürich, Switzerland, for a partially automated spatial analysis to understand densification patterns and transport energy usage for sustainable development. Densification patterns [4] are derived from data on the distribution of building densities and land uses. Transport energy was derived from data on traffic volume and speeds. Resulting insights are pertinent to achieving more sustainable cities because housing, public and commercial services and transport account for more than half of all energy usage in cities [5]. Furthermore, the data allows for questions on (i) what conditions are required for sustainable development understood through densification patterns or transport energy usage, (ii) which direct or indirect relationships can be observed when comparing spatial configurations across different cases, and (iii) what is the best way to collect, prepare and automate similar data for comparison across context and case.

Most of the data prepared are from publicly available open-source databases wherever possible. The exceptions to this rule are the unique, validated hand-drawn axial maps from which urban structure is calculated and the proprietary data on transport flow. Data proxies or alternatives to proprietary or unobtainable data required to understand urban structure and form are provided. For example, the building density data for Zürich was approximated through open-source data as it was not available to the public. There is no significant observable difference in the provided proprietary data from Bergen versus that proxy data built by the authors on Zürich.

Each city studied requires a data package, combining primary (i.e. self-drawn axial maps) and secondary data (i.e. building plot outlines, property characteristics) obtained from public authorities and open-source data. The spatial data consists of the object (a point, line or polygon with x-y coordinates) and an attached attribute table consisting of relevant information to the spatial object (i.e., shape, size, ownership information etc.). The unique data for urban structure are axial lines drawn by the researcher as an overlay on existing maps and transformed into first-hand empirical data in the form of a georeferenced shapefile for each data input for both cities. The shapefile format (*shp) is commonly used on GIS platforms. There are five types of data used for each case.

1. **Urban structure** represented as line segments (from axial drawing) including attribute data such as network values of closeness and betweenness (see Fig. 1a and d); and

**Fig. 1.**
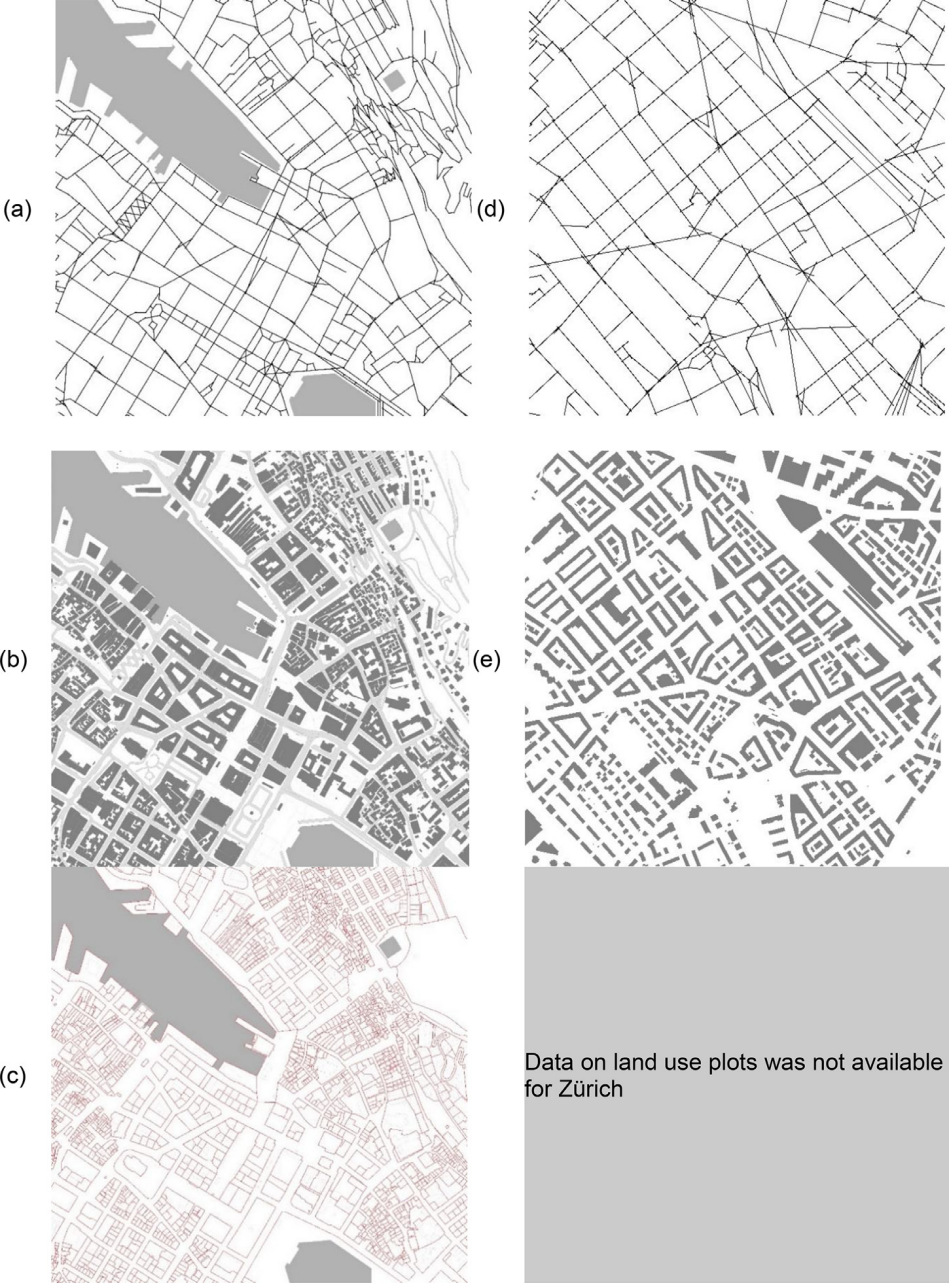
1 × 1 km visual representation of data types for Bergen, Norway (left) and Zürich, Switzerland (right). Fig. 1a, d show axial line maps, 1b and 1e show building maps, and 1c shows a land-use plot map.
2. **Urban form** is represented as polygons (see Fig. 1b and e) with building attribute data such as dimensions, age and functions.

These data are the key independent variables for both cases. Extra information that permits us to identify densification patterns is represented by:

3. **Building density** is calculated via the dimensions and spatial location of the land use plots (see Fig. 1c). This is only available for Bergen due to a lack of proprietary data.;

Relating urban structure and form to transport energy usage are represented by:

4. **Road centre lines** containing dimensions and spatial location of the network of roads, streets, paths and alleyways; and
5. **Transport use**, the amounts of traffic and maximum speeds on the roads and streets to calculate transport energy usage.

The five data types are explained here in detail.

• **Urban structure**, showing the space between buildings, is partially represented as line segments for Bergen [200707_BERGEN_SS.shp] and Zürich [191014_ZÜRICH_SS.shp]. The data is stored in georeferenced vector maps. The files contain single, straight lines between two geo-coordinates that intersect or overlap each other and cover all convex urban spaces using the fewest, longest lines determined by the line of sight (see Fig. 1a and d). The sample total for Bergen is 35,304 segment lines created from 8534 axial lines and 43,443 segment lines derived from 9398 axial lines for the study area Zürich. The relevant (tabular) attribute fields attached to the shapefile objects are the metrics of betweenness and closeness calculated using the open-source software program depthmapX (https://spacegroupucl.github.io/depthmapX/). The metric betweenness shows the likelihood of a street segment to be part of a route and is found with Angular Choice calculations. This takes into account cognition and wayfinding and explains the potential of movement through a network of streets. For example, most trips taken within a street network in a given radius will go through the street segment with the highest choice value. The metric closeness demonstrates the likelihood of a street segment as a destination and is calculated via Angular Integration. This expands on current approaches to understanding networks through the hierarchy (speed and volumes of streets) and accounts for the occurrence of movement as destination potential of a street segment within a given network. For example, a street segment on which the shortest path between most pairs of segments within a given radius falls on will have a high value [6]. Both metrics are calculated at the neighbourhood (500m radius) and citywide (5000m) scale. The choice for 500 or 5000 meters as the radius is related to the logic of space assumption [2,3], which suggests that these dimensions are where pedestrians or car users would make a cognitive choice that determines their routes.

These fields are named:

• 'NACH500′ categorised as 'C_500′; values for Normalised Angular Choice or NACH (representing 'betweenness') on a 500 m metrical radius for each segment;
• 'NACH5000′ categorised as 'C_5000′; values for NACH at a 5000 m radius for each segment;
• 'NAIN500′ categorised as 'I_500′; Normalised Angular Integration or NAIN (representing 'closeness') on a 500 m radius for each segment; and
• 'NAIN5000′ categorised as 'I_5000′; values for NAIN at a 5000 m radius for each segment.

The above fields are categorised into three value types (low, medium, and high). The last step of preparing the data on spatial configurations is to aggregate all betweenness (named 'IAGGR') values and all closeness (named 'CAGGR') values to capture how street segments simultaneously perform across two levels of scale. The categorisation and aggregation procedures are described in detail in the next section.

• **Urban form** is represented through polygons that represent the dimensions and spatial location of the buildings and related land use and building density data (when available). The files within the dataset are named [BERGEN_BLD.shp] for Bergen and [191104_ZÜRICH_BLD_NO_HSQ.shp] for Zürich. The sample total for Bergen is 22,548 and for Zürich 22,118 buildings. The relevant secondary data are not equally available for both cases. The information about the land uses for Zürich was incomplete and not open-source. However, the missing information was added manually by checking Google Earth and Google Street View.

These files contain eight fields:

• 'TYPEKODE': shows values of land use. Each land use has a unique three-digit code associated with legal status, stored in this attribute field (see Table 1). A custom script is used to derive the secondary data from this attribute field relevant to this research, namely the ratio between the urban activities performed. In addition, a distinction is made between amenities, offices, and housing for each building. This script is described in the next section.

**Table 1:** Attribute codes and functions.

Attribute code	Function	Type of activity
100-199	Residential	Housing
200-249	Industrial	Offices
300-349	Office/business	Offices
400-449	Transport/communication	Amenities
500-549	Hotel/Restaurant	Amenities
600->	Remaining amenities	Amenities
• 'F_ETASJER': contains the numerical values of the number of building floors;
• 'BRUKSAREAL': values on the area of the building footprint; and
• 'F_BOENHETE': the number of housing units.

In addition, attribute fields for understanding urban form in relation to land use functions for both cases are derived from the above four fields. These attribute values indicate the degree of functional mix or how diverse activities are located or distributed in one particular location. These fields are:

• 'AMENITIES': the percentage of total floor space used for amenities (e.g. transportation, hotels, restaurants, and other amenities);
• 'OFFICES': the percentage of total floor space used for offices and business and industrial functions.
• 'HOUSING': the percentage of total floor space used for residential purposes; and
• 'MXI': the distribution of the above fields AMENITIES, OFFICES, and HOUSING categorised based on the Form Syntax framework [7]. Here, Van den Hoek's [8] terminology was used and corresponded with Dovey and Pavka's 'live' (housing), 'work' (offices) and 'visit' (amenities) classification [9]. The same four attribute fields as for Bergen were added manually for Zürich via open-source online information, rather than calculated via data as was done for the case of Bergen.
• **Building density** helps to understand how urban form is utilised and calculated with the file [200107_BERGEN_PLOTS.shp]. This shapefile is obtained from the municipality of Bergen and contains the geometry of the cadastral pattern, i.e. the plot size and shape on which the buildings stand (see Fig. 1c). The plot sizes are used for calculating building density explained in the next section. However, as open-source data for land for Zürich was not available, this applies to the densification patterns in the case of Bergen only.
• **Road centre lines** are the common feature used for linking transport energy data with outputs of the spatial analysis via depthmapX. The files are named [180906_BERGEN_RCL.shp] for Bergen and [180906_ZÜRICH_RCL.shp] for Zürich. These files contain data contains road centre line data that comes from OSM. It includes the dimensions (length, width and type of roads) and the spatial location of the road and street network in the form of polylines. The key attribute field used is 'maxspeed'. It represents the maximum traffic speed (km/h) on a particular road.
• **Transport use**, which shows the capacity and volume of traffic on the roads analysed, are found in the files [181004_ÅDT_Hordaland.shp] for Bergen and [Macroscopic_Assignment_Model_ZH_link.shp] for Zürich.These files attach attribute data that show traffic volume and flow to road centre lines. These data are proprietary and sourced from the Norwegian Public Road Administration and the Institute for Transport Planning and Systems at ETH Zürich. Relevant attribute fields showing Annual Average Daily Traffic (AADT) for each road segment as the fields' ÅDT_tota' for Bergen and 'VOLVEHPR∼2′ for Zürich. Traffic volume from Bergen is measured. From Zürich, this data is simulated [10]. These fields are used to calculate energy usage by cars for each road/street segment. Detailed calculations are described in the next section.

As described, there are some discrepancies in data across both cases, especially for building density and transport use. This is due to differences in data collection systems and legislation in both countries. In addition, access to data is challenging for Zürich. In Bergen, access to data also resulted in more precise building density values and transport use. In Zürich, data had to be supplemented manually for land use or omitted for plot size. Cross-case comparisons for densification patterns were therefore not possible. On the other hand, in Zürich, data could be simulated in the case of transport use. Hence, comparisons for transport energy usage were possible.

## 2 Experimental Design, Materials and Methods

**Fig. 2.**
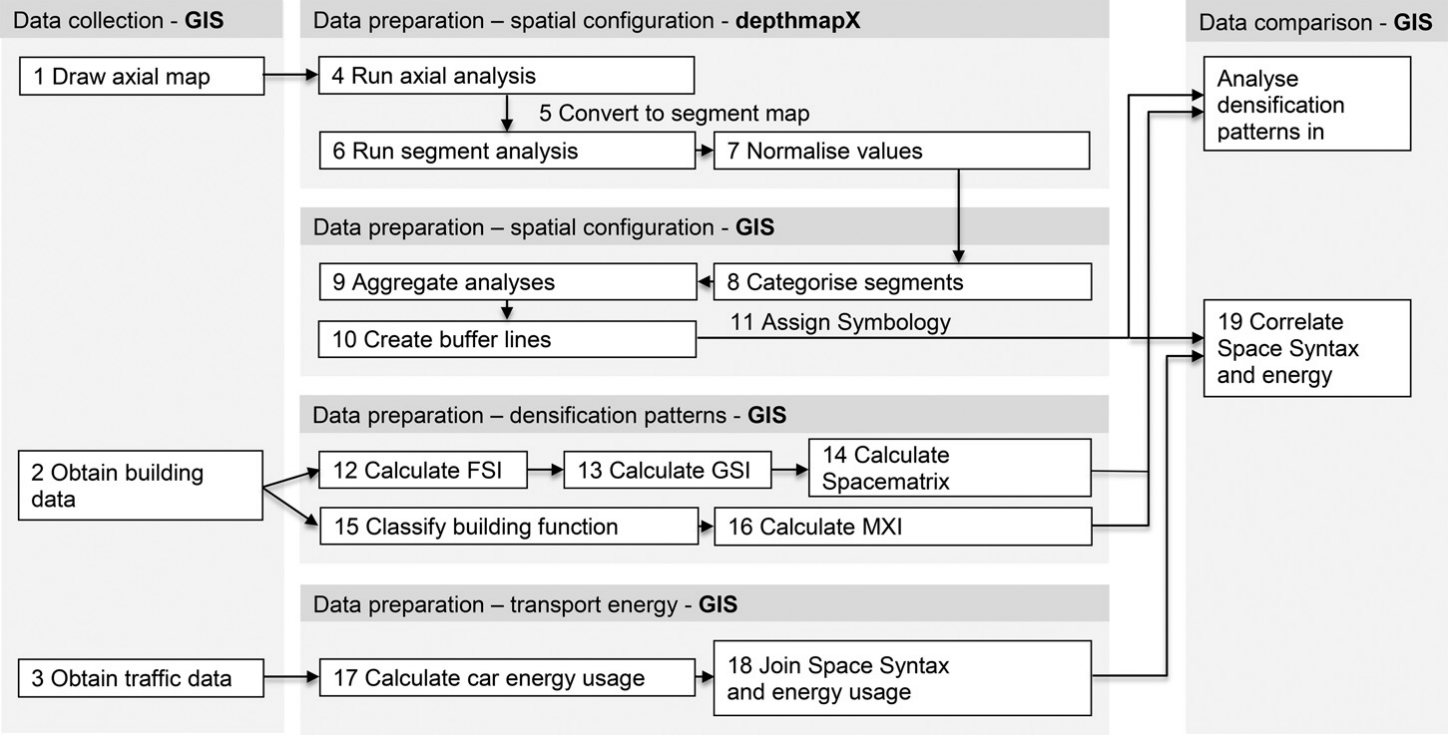
Schematic diagram of the workflow.

The data above was processed in an experimental workflow (see Fig. 2) designed to combine GIS with the open-source program depthmapX used to calculate spatial configurations and to facilitate multiple iterations of data processing and analysis with various inputs from both cases. The GIS software used here is ESRI® ArcMap version 10.4.1 for Desktop. The workflow has three stages: data collection, preparation, and comparison. The 19 steps proposed contributes to novel ways to identify and compare spatial configurations through efficiently aggregating and comparing data that are both spatial and non-spatial from different base units (i.e. levels of scales) and different sources. For example, the application of a buffer operation to better compare building level data with street network values is a combination of spatial and non-spatial data. In addition, the authors have shared how to automate the workflow such that internal validity is assured when comparing cases or when updates of data are available.

### 2.1 Data Collection and Combination

The three steps of combining the data previously described are;

#### 2.1.1 Steps 1 – 3

1. **Drawing axial maps**; a new shapefile was created in ArcMap and drawing straight lines by hand over an existing georeferenced map. Each line segment follows the Space Syntax [11] rules of thumb. Each line indicates the longest lines of sight, using the fewest lines possible to cover and connect all convex accessible spaces in the urban system between the physical objects making all direct line-to-line connections (i.e. each line must intersect and/or overlap another line). When lines intersect, one line of movement ends, and another continues. There are no continuous corners drawn. In the event of overlapping or 'unlinks' such as a viaduct, underpass or tunnel, a separate shapefile is created to mark points the overlap occurs. Where a viaduct crosses over another road, this is shown as two separate and non-intersecting lines. The amount of lines intersecting on each line affects its relational value to the entire network of lines, and hence the value of the metrics returned with the depthmapX analysis. Both files are exported as DXF file format and analysed with depthmapX before being exported back to ArcMap.
2. **Obtaining building data**; these are georeferenced spatial data as shapefiles from the local authorities (Bergen Kommune) and, in the case of Zürich from OSM. The municipality stores detailed metadata such as functional use, number of floors, number of housing units, and information on the year of construction, heritage status and others for each building. Where information was incomplete, manual checking via Google Maps was done. Plot data for building density is contained in a separate shapefile.
3. **Obtaining traffic data**, the objects in the shapefiles are road centre lines representing the geometry of the streets and roads retrieved from online sources (OSM). For the case of Bergen, measured traffic data and information about the maximum traffic speed was obtained as attribute data from the Norwegian Public Road Administration (SVV). For the case of Zürich, a calibrated traffic model created by the Institute for Transport Planning and Systems at ETH Zürich was used [10].

### 2.2 Data Preparation

Due to the different platforms for analysis and the different expected outcomes (densification patterns or transport energy usage), a series of data preparation steps are required that differ in calculations or aggregation. Step 4–11 performed in depthmapX prepare axial maps for analysis to obtain spatial configurations. Building data is prepared in Steps 12–16 to understand densification patterns. In Steps 17,18, transport energy usage is calculated, and the values to be compared are joined. Steps 8–18 are done in ESRI® ArcMap. Steps 15, 16 and 18 have been automated. See the next section for details.

#### 2.2.1 Steps 4 – 7: Depthmapx Operations for Spatial Configuration

Within the depthmapX interface, after importing the axial map in DXF format created in ArcMap, the graph analysis menu is used to run axial analysis. Input for the menu includes a radius of *n* to obtain results for global integration analysis (from all lines to all others, see [11], p. 48,49 for an explanation of the calculation of global integration) and a radius of value 3 to get the local integration analysis. These analyses must be done before converting the map to a segment map.

Segment maps are generated using the function of 'convert active map'. depthmapX will split up all axial lines into separate segments where they cross each other. Next, the function of 'Run angular segment analysis' opens a dialog window where 'Metric' is chosen as radius type. For the neighbourhood and citywide scales, 500 and 5000 metres radii are entered, respectively. The base unit of the georeferenced map on ArcMap is in meters. Hence, the values are entered without unit conversion. Next, values from the previous steps are normalised to obtain NACH500 and NACH5000 (for Normalised Angular CHoice) and NAIN500 and NAIN5000 (for Normalised Angular INtegration) with the following formulae. These values are joined to the segment map file as four new attributes. The normalised values are obtained by populating these attribute columns with a normalisation formula. The calculated values for Angular Choice were normalised using the following formula for each radius [12]:

$$\text{NACH}\;=\;\frac{\text{log}(\text{Choice}\;\left(r\right)\;+\;1)}{\text{log}(\text{Total}\;\text{depth}\;\left(r\right)\;+\;3)}$$

The non-normalised values are auto-generated in depthmapX under the following fields' T1024 Total Depth', 'T1024 Node Count' and 'T1024 Choice'.

Following this, the following formula is entered into the “Replace values” interface for both radii:

**Table:** 

log(value("T1024 Choice R500/R5000 metric")+1)/log(value("T1024 Total Depth R500/R5000 metric")+3)

The values for NACH500 range from 1.079171 to 5.59329 for Bergen and from 1.0413927 to 5.5103316 for Zürich. For NACH5000, values range from 1.079171 and 8.116694 for Bergen and from 1.0413927 to 8.3752794 for Zürich.

For integration, normalisation values are obtained from [11]:

$$\text{NAIN}=\;\frac{\sqrt[{1.2}]{\text{Node}\;\text{count}\;(r)}}{\text{Total}\;\text{depth}\;\left(r\right)+2}$$

The following formula is entered into the “Replace values” interface for both radii:

**Table:** 

(value("T1024 Node Count R500/R5000 metric)^(1.2)/(value("T1024 Total Depth R500/R5000 metric")+2)

The values for NACH500 range from 0.754693 to 2.848512 for Bergen and from 1.0430746 to 2.8643966 for Zürich. For NACH5000, values range from 2.474288 and 3.672265 for Bergen and from 3.0674546 to 3.9541197 for Zürich.

To export the values above to a GIS-compatible format, it is saved as a MIF file which maintains its georeferencing. The axial and segment maps are exported as separate MIF files and then converted to the ESRI® Shapefile format (SHP) using QGIS or ArcGIS.

#### 2.2.2 Steps 8 – 11: ArcMap Operations for Spatial Configurations

Resulting values from steps 4 – 7 are categorised into low (value of 1), medium (value of 2) and high (value of 3) values for each attribute following the 'natural break' method [13,14] via the Symbology menu. These values are contained in the attribute tables of the relevant shapefiles.

**Fig. 3.**
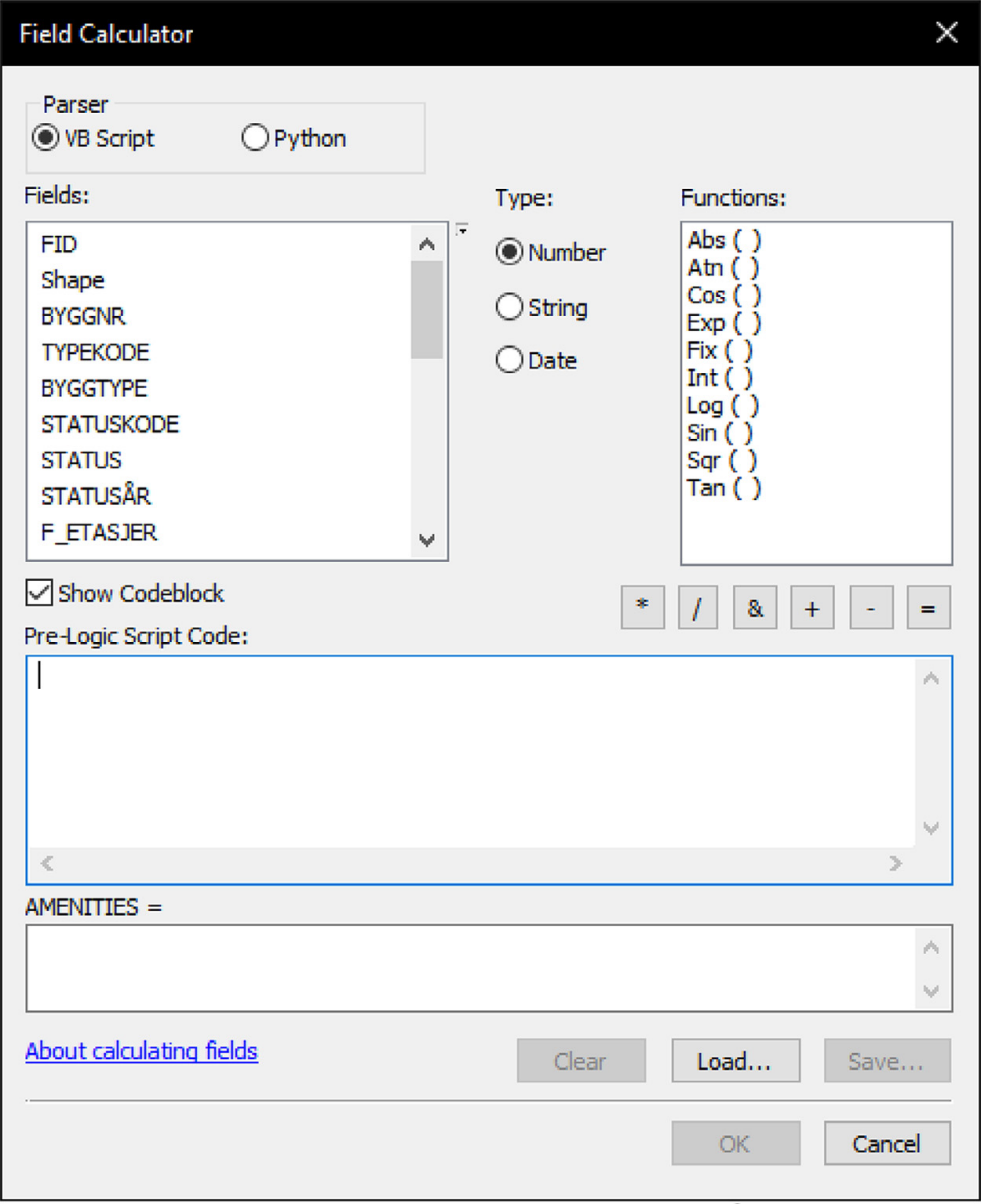
Field Calculator GUI in ESRI® ArcMap.

The values are categorised using the Visual Basic script in Field Calculator in ArcMap. This tool calculates the values of a field for a feature class [15] (see Fig. 3). This can be an arithmetic formula or more advanced Visual Basic or Python scripts, which can be entered in the "Codeblock". VB Script is used for consistency and in relation to the relatively simple operations required. The attribute field(s) to be queried can be selected on the left. The data type is assigned in the middle, and on the right, different mathematical functions can be added. Field Calculator uses (conditional) if-statements in the Codeblock input field under the Pre-logic script code window.

The calculations are assigned to a new categorised attribute (randomly named 'a' in this article). The script used is:

**Table:** 

[CATEGORISED_ATTRIBUTE_NAME] = a

dim a if [UNCATEGORISED_ATTRIBUTE_NAME] < "(threshold low-medium)" then a = "1" elseif [UNCATEGORISED_ATTRIBUTE_NAME] > "(threshold medium-high)" then a = "3" else a = "2" end if

Here, 'UNCATEGORISED_ATTRIBUTE_NAME' is the name of the attribute containing the uncategorised normalised choice and integration values: 'NACH500′, 'NACH5000′, 'NAIN500′, and 'NAIN5000′. 'CATEGORISED_ATTRIBUTE_NAME' is the new attribute fields containing the resultant categorised values' C_500′, 'C_5000′, 'I_500′, and 'I_5000′. The threshold low-medium and medium-high are the numerical thresholds between low and medium and medium and high values for each of the four Space Syntax measures, calculated based on the statistical distribution of natural breaks. The threshold values will be different for each study case since it depends on the relational values of each segment to the street network under investigation.

To better analyse normalised values related to other variables, a matrix combining high and low choice values needs to be produced. This is done by aggregating the categorised low scale and the high scale values with another Visual Basic script to populate an attribute field 'CAGGR' with values based on 'NACH500′ and 'NACH5000′.

For angular choice, the categorised low scale attribute is named 'C_500′, and the categorised high scale attribute is named 'C_5000′. The script returns a two-letter string indicating a low (L), medium (M) or high (H) value for citywide scale and neighbourhood scale, respectively (see Table 2).

**Table 2:** Aggregating low radius and high radius.

		Angular choice with low radius (R = 500 m)		
		Low	Medium	High
Angular choice with high radius (R = 5 000 m)	Low	LL	LM	LH
	Medium	ML	MM	MH
	High	HL	HM	HH

The script for Aggregated Angular Choice is:

**Table:** 

[CAGGR] = a
Pre-logic script code:
dim a
if [C_5000] = 3 and [C_500] = 3 then
a = "HH"
elseif [C_5000] = 3 and [C_500] = 2 then
a = "HM"
elseif [C_5000] = 3 and [C_500] = 1 then
a = "HL"
elseif [C_5000] = 2 and [C_500] = 3 then
a = "MH"
elseif [C_5000] = 2 and [C_500] = 2 then
a = "MM"
elseif [C_5000] = 2 and [C_500] = 1 then
a = "ML"
elseif [C_5000] = 1 and [C_500] = 3 then
a = "LH"
elseif [C_5000] = 1 and [C_500] = 2 then
a = "LM"
elseif [C_5000] = 1 and [C_500] = 1 then
a = "LL"
end if

Next, each segment line receives a buffer. This is done through the Buffer Tool in ArcMap to a radius of 35m and with a round bevel. Each buffer is merged with identical categorised aggregated choice values from the previous step. The Clip Tool is then used to remove the overlap of the buffers of various categories. To visualise the outputs, an optional step is introduced to create a map in the workflow (see Fig. 2, Step 11). The Symbology menu determines the choice of colour, line density, and iconography to customise which attributes are visualised and how. Desired settings can be saved to a separate file (called layer file, in LYR format) for repeating across cases.

#### 2.2.3 Steps 12 – 16: Densification Patterns at the Building Level

These steps result in values of floor space index (FSI) and ground space index (GSI), which provides insights into building density values using the Spacematrix method [16]. First, the plot data (specifically the attribute column containing area size) is joined to the building data through the Spatial Join tool. This adds an attribute field in the building shapefile that contains the plot area size. Next, a new attribute column 'FSI' is added and populated using the following formula:

$$\text{Floor}\;\text{Space}\;\text{Index}\;(\text{FSI})\;=\;\frac{\text{floor}\;\text{space}\;\text{area}}{\text{area}\;\text{of}\;\text{the}\;\text{plot}}$$

where floor space area is the total building's floor space (all floors) is divided by the total area of the plot. This is achieved in the Field Calculator by entering the formula above.

Next, GSI can be derived from the geometry of the plot and the building by the following formula:

$$\text{Ground}\;\text{Space}\;\text{Index}\;\left(\text{GSI}\right)\;=\;\frac{\text{ground}\;\text{space}\;\text{area}}{\text{area}\;\text{of}\;\text{the}\;\text{plot}}$$

Where ground space area is the attribute column containing the building footprint. This yields values ranging between 0 (unbuilt) and 1 (fully built).

A basic script is made in the Field calculator to classify buildings from (A) to (I) in a new attribute field called 'SPACEMATR'. The classification is based on GSI values where 0.15 and 0.25 [7] were taken as thresholds and the number of floors:

**Table:** 

[SPACEMATR] = a
Pre-logic script code:
dim a if [GSI] < 0.15 and [F_ETASJER] < 3 then a = "A" elseif [GSI] < 0.15 and 3 <= [F_ETASJER] < 7 then a = "D" elseif [GSI] < 0.15 and [F_ETASJER] >= 7 then a = "G" elseif [GSI] >= 0.25 and [F_ETASJER] < 3 then a = "C" elseif [GSI] >= 0.25 and 3 <= [F_ETASJER] < 7 then a = "F" elseif [GSI] >= 0.25 and [F_ETASJER] >= 7 then a = "I" elseif 0.15 <= [GSI] < 0.25 and [F_ETASJER] < 3 then a = "B" elseif 0.15 <= [GSI] < 0.25 and 3 <= [F_ETASJER] < 7 then a = "E" elseif 0.15 <= [GSI] < 0.25 and [F_ETASJER] >= 7 then a = "H" else a = "X" end if

Building functions are related to densification patterns. This is classified in Table 1 as the attributes amenities, offices and housing. Here, three Field Calculator scripts are executed for each attribute to convert the values to their corresponding MXI categorisation. Unfortunately, each building may contain only one function code. For example, in a high rise building with multiple units, the code attached may indicate offices, but if the polygon feature has values in the other attributes of 'F_BOENHETE', which lists the number of housing units in each building, then that object will be listed as a bi-functional building. The following scripts will be determined using the attribute 'F_ETASJER', the number of floors, the ratio of 'Amenities' vs 'Housing' vs 'Office'.

For 'AMENITIES', the script is:

**Table:** 

[AMENITIES] = a
Pre-logic script code:
dim a if ((([TYPEKODE] > "200") and ([TYPEKODE] < "300") and ([F_BOENHETE] = "0")) or (([TYPEKODE] > "320") and ([F_BOENHETE] = "0"))) then a = [F_ETASJER] elseif ((([TYPEKODE] > "200") and ([TYPEKODE] < "300") and ([F_BOENHETE] > "0")) or (([TYPEKODE] > "320") and ([F_BOENHETE] > "0"))) then a = "1" else a = "0" end if

For [OFFICES], the script is:

**Table:** 

[OFFICES] = o
Pre-logic script code:
dim o if (([TYPEKODE] < "320") and ([TYPEKODE] > "300" and ([F_BOENHETE] = "0"))) then o = [F_ETASJER] elseif (([TYPEKODE] < "320") and ([TYPEKODE] > "300") and ([F_BOENHETE] > "0")) then o = "1" else o = "0" end if

For [HOUSING], the script is:

**Table:** 

[HOUSING] = h
Pre-logic script code:
dim h if [TYPEKODE] < "200" and [F_BOENHETE] > "0" then h = [F_ETASJER] elseif [TYPEKODE] > "200" and [F_BOENHETE] > "0" then h = ([F_ETASJER] - ([AMENITIES] + [OFFICES])) else h = "0" end if

The mix and diversity of building functions are calculated by looking at the ratio between 'AMENITIES', 'OFFICES', and 'HOUSING', resulting in the Mixed-Used Index' MXI' based on the method, see [8,9]. Each value MXI is categorised with a combination of letter codes (see Table 3) according to thresholds established in previous research [7], p. 78.

**Table 3:** Letter codes for Mixed-Use Index categorisation.

Letter code	Functionality
A	Mono-functional, amenities
O	Mono-functional, offices
H	Mono-functional, housing
HA	Bi-functional, housing and amenities
HO	Bi-functional, housing and offices
AO	Bi-functional, amenities and offices
TH	Triple-functional, predominantly housing
TA	Triple-functional, predominantly amenities
TO	Triple-functional, predominantly offices

[MXI] is calculated by the following script:

**Table:** 

[MXI] = m
Pre-logic script code:
dim m if [HOUSING] < 0.2 and [HOUSING] > 0.05 and [AMENITIES] > 0.05 and [OFFICES] > 0.05 then m = “TH” elseif [AMENITIES] < 0.2 and [AMENITIES] > 0.05 and [HOUSING] > 0.05 and [OFFICES] > 0.05 then m = “TA” elseif [OFFICES] < 0.2 and [OFFICES] > 0.05 and [HOUSING] > 0.05 and [AMENITIES] > 0.05 then m = “TO” elseif [HOUSING] > 0.05 and [AMENITIES] > 0.05 and [OFFICES] < 0.05 then m = “HA” elseif [HOUSING] > 0.05 and [OFFICES] > 0.05 and [AMENITIES] < 0.05 then m = “HO” elseif [AMENITIES] > 0.05 and [OFFICES] > 0.05 and [HOUSING] < 0.05 then m = “AO” elseif [HOUSING] > 0.95 and [AMENITIES] < 0.05 and [OFFICES] < 0.05 then m = “H” elseif [AMENITIES] > 0.95 and [OFFICES] < 0.05 and [HOUSING] < 0.05 then m = “A” elseif [AMENITIES] < 0.05 and [OFFICES] > 0.95 and [HOUSING] < 0.05 then m = “O” else m = “S” end if

**Fig. 4.**
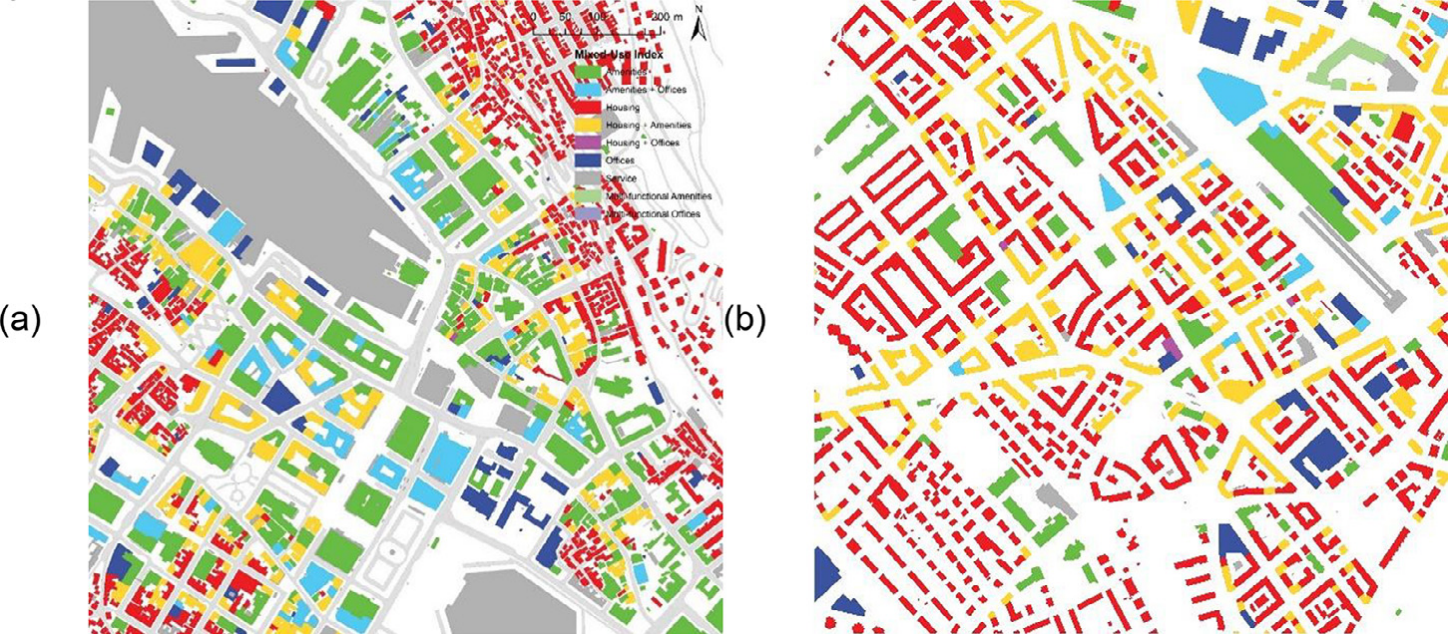
1 × 1 km excerpts of the Mixed-Use Index (MXI) map for Bergen (left) and Zürich (right).

Fig. 4 below shows an example of the MXI results for Bergen and Zürich.

The above steps are sufficient to generate insights into densification patterns concerning urban structures and forms. Preliminary results from the data show that energy usage in cities correlates with its spatial configuration. The denser and more compact the city, the more concentrated and efficient the expected energy usage. Compact cities, where highly integrated transportation networks connect local centres with high building and function densities, are more energy-efficient and sustainable [1,4].

#### 2.3.4 Steps 17 – 18: Transport Energy Usage

Transport energy usage is calculated from traffic speed, traffic volume and average energy consumption from cars. This step uses the Road Centre Line (RCL) as the object combining road geometry and traffic data. The total energy usage of car traffic on each road segment is represented as kWh per day. The calculation is based on the attribute fields containing the maximum allowed traffic speed and the total number of cars travelling daily across a segment expressed as the Annual Average Daily Traffic (AADT).

The calculation is derived from [5]:

Etot=(numberofcarsperday)·(2.08·(trafficspeed)3+400·(trafficspeed))

A new attribute field [KWH] is populated using the following expression in Field Calculator:

**Table:** 

[KWH] = (2.08 * [freespeed] * [freespeed] * [freespeed] + 400 * [freespeed]) * [carvolume])

Next, the processed Space Syntax data (Steps 4 – 7) are joined to the energy usage results contained on the RCL features (Step 17) through a one-to-one Spatial Join. This is based on proximity, whereby the nearest/closest line segment will inherit the normalised values. This step is required to run a correlation analysis for data comparison. The RCL feature class inherits all data joined in its attribute table when the Spatial Join tool is executed. This attribute is then exported as a table (e.g. CSV or DBF format) for statistical correlation.

One of the goals of the workflow described is to verify whether the urban structure and form influence transport energy usage. For example, historic city centres tend to have highly integrated spatial structures (this can be quantified by betweenness and closeness) and are highly suitable for walking and cycling [1,7]. This relationship is verified via correlating spatial configuration data (from depthmapX) using the Space Syntax methods [2,3,17]. The data is compared statistically with the values generated in Step 17 using a bivariate correlation, Pearson correlation coefficients and two-tailed test significance in SPSS (see for results [1], Table 4).

### 2.4 Workflow Automation

The above steps for data collection and data preparation are then automated. This ensures an efficient repetition of the necessary input processes, cross-platform data translation, data aggregation, and combination calculations for analysis. This partial automation is done by designing data operations specified through a Visual Programming Language (VPL) [18] via the ModelBuilder^TM^ in ESRI® ArcGIS. The workflow shared can support fellow researchers and planning professionals in adopting the proposed method for analysing urban form and structure. Making the steps of the workflow explicit can also support the decision-making processes of policymakers. This can increase understanding of how cities function and develop them more sustainably.

**Fig. 5.**
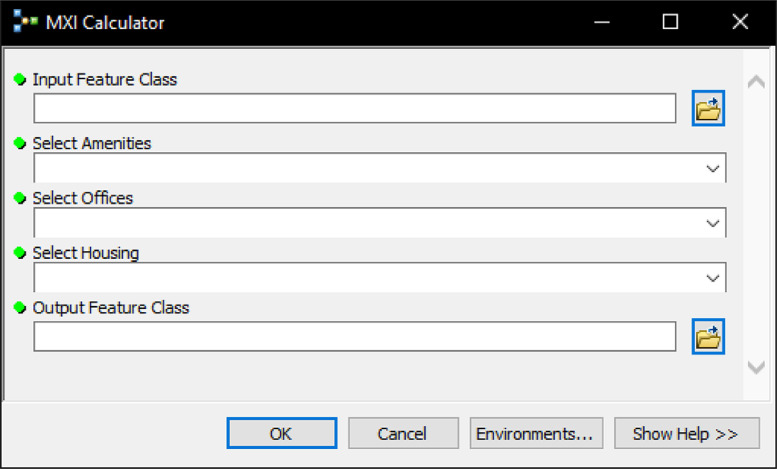
Graphical User interface of the Mixed-Use Index Calculator.

The models designed and tested are provided as an ArcGIS Toolbox file, which is a container for the various tools and steps of operations within ArcGIS (see Data preparation section, Steps 8 – 19). The Toolbox file allows for the automation (or parts of) the scientific workflow that requires a repetition of tasks and/or standardisation of analytical procedures. Users do not need to specify additional steps or perform any geocomputational tasks manually. The end-user is presented with a Graphical User Interface (GUI) (see Fig. 5). Relevant input parameters and variables can be selected from a drop-down menu in this interface. When exported and made usable for other researchers who might have limited resources or computing capacities, it reduces potential complications or human error when inputting multiple datasets and increases the internal validity of the research.

**Fig. 6.**
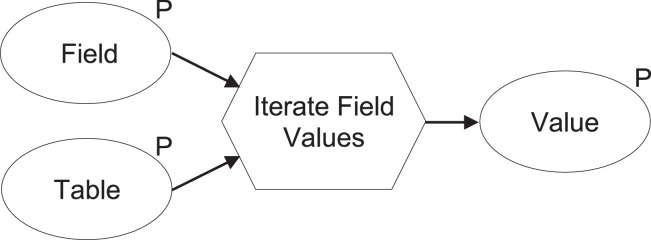
Iterator model.

**Fig. 7.**
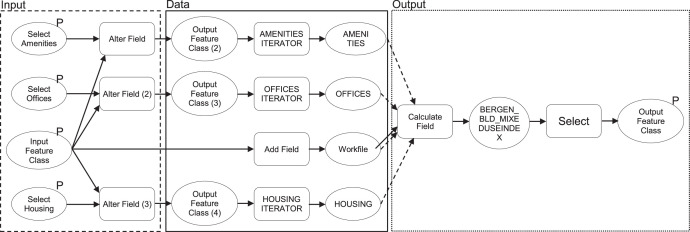
Mixed-Use Index Calculator model.

The file [REK_Urban_Analysis_Toolbox.tbx] consists of standard, pre-programmed operations combined by the authors into complex models. Within the ModelBuilder environment, a model is comprised of four elements, (i) geoprocessing tools and (ii) variables linked by (iii) connectors to indicate the direction of processing and can be combined as (iv) groups as required. For the data presented, these elements are used (see Figs. 6 and 7);

• Tools to run specific tasks include adding a buffer or calculating values shown as rounded rectangles. Special tools, including Iterators, are used here to repeat or loop the operations within a model and are shown as a hexagonal node.
• Variables for inserting input or (derived) output such as shapefiles or data shown as oval nodes and indicated with parameter or P when editable. Output parameters allow users to indicate their desired file name and location for saving. Variables are also shown as dependencies, temporary files after each task before being linked to another operational step.
• Connectors link variables to tools depending on the required process as uni-directional arrows. They indicate how the model should flow. These can be linked and unlinked as required.
• Groups show a combination of the above elements that are collapsible when needed to represent a simplified model.

Next, two custom models, the iterator and the MXI calculator models, are described. The first is a custom model built to facilitate data preparation, and the latter allows for outputs to calculate the distribution of land uses needed for Step 16.

### Iterator Model

Each model may contain only one iterator node (see Fig. 6). However, some models require more than one instance of an iterator to perform the desired tasks. For example, the following model can be saved as a custom tool and inserted within a composite model. For the data presented, the iterator model is required to convert data for urban form (i.e., numerical code data for the categories of functions such as amenities, offices and housing) according to building density data (i.e., the ratio of floor space for a specific function to total floor space) to allocate the required letter code categories to indicate the mix of functions via the Mixed-Use Index method (see Table 3).

For example, the input Field would be the calculation of the ratio of floor space for offices, and the input Table would be a selection of codes belonging to buildings with office functions. The iterator tool will be asked to calculate how much of a particular building would belong as offices and cycle it again through all building features in the file until each feature is assigned a corresponding value. Grouping the input, output and iterator together allow it to be saved as a reusable iterator model (rounded rectangle) for building a more complex composite model with only minimal changes to input and output parameters. This is used in the data presented for all three functions within the mixed-use index model (see Fig. 7).

### Mixed-Use Index Calculator

A key contribution to data preparation (Steps 12 – 16) requires identifying types of functions within each building and coded based on the ratio of floor space per function to total floor space per building. The calculation is complex and requires a composite model, i.e., the custom Mixed-Use Index (MXI) Calculator model created by the authors (see Fig. 7). The left part (in the dashed rectangle) of the model is where the input parameters are specified. The middle part (in the solid rectangle) prepares the data. Finally, the right part (in the dotted rectangle) calculates MXI and generates the output. The model consists of nine tools, four input variables, eight interim output variables, and a final output variable that indicates the MXI data as strings, i.e. letters or text (see Table 3) assigned to each feature within the urban form dataset. In addition, three custom iterator models (built as stated above) – the amenities, offices and housing iterators – enable the MXI Calculator to generate output for connecting urban form and building density data to understand densification patterns automates four steps (Steps 12 – 16) to be repeated per case in a single step.

## Ethics Statements

The above works do not contain information from human subjects, animal experiments or data collected from social media platforms.

## Funding

This work was supported by the Norwegian Research Council (NFR, grant No 261179), JPI Urban Europe (E.U. Horizon 2020 research and innovation programme, grant agreement No 693443), NWO, DETEC and RCN.

## CRediT authorship contribution statement

**Remco Elric de Koning:** Conceptualization, Methodology, Software, Validation, Formal analysis, Investigation, Data curation, Writing – original draft, Writing – review & editing, Visualization. **Rogardt Heldal:** Writing – review & editing, Supervision. **Wendy Tan:** Writing – review & editing, Supervision.

## Declaration of Competing Interest

The authors declare that they have no known competing financial interests or personal relationships that could have appeared to influence the work reported in this paper.

## Data Availability

HVL_PhD (Original data) (github).

## References

[1] de Koning R.E., Tan W.G.Z., van Nes A. (2020). Assessing spatial configurations and transport energy usage for planning sustainable communities. Sustainability.

[2] Hillier B. (1996).

[3] Hillier B., Hanson J.M. (1984).

[4] de Koning R.E., Roald H.J., van Nes A. (2020). A scientific approach to the densification debate in bergen centre in Norway. Sustainability.

[5] MacKay D. (2009).

[6] Rashid M. (2017).

[7] Ye Y., Yeh A., Zhuang Y., Van Nes A., Liu J. (2016). Form syntax" as a contribution to geodesign: a morphological tool for urbanity-making in urban design. Urban Des. Int..

[8] van den Hoek J., Stolk E., Brömmelströet M.T. (2009). New Towns for the 21st Century: the Planned vs the Unplanned City.

[9] Dovey K., Pafka E. (2017). What is functional mix? An assemblage approach. Plan. Theory Pract..

[10] Horni A., Nagel K., Axhausen K. (2016).

[11] van Nes A., Yamu C. (2021).

[12] Hillier B., Yang T., Turner A. (2012). Normalising least angle choice in depthmap and how it opens new perspectives on the global and local analysis of city space. J. Space Syntax.

[13] ESRI, Data classification methods. Abailable at: https://pro.arcgis.com/en/pro-app/latest/help/mapping/layer-properties/data-classification-methods.htm, 2021. Accessed December 27, 2020.

[14] Longley P.A., Batty M. (1996).

[15] ESRI, Calculate Field. Abailable at: https://desktop.arcgis.com/en/arcmap/latest/tools/data-management-toolbox/calculate-field.htm, 2021. Accessed December 27, 2021.

[16] Rådberg J. (1988). Statens Råd för Byggnadsforskning.

[17] Hillier B., Penn A., Hanson J., Grajewski T., Xu J. (1993). Natural movement: or, configuration and attraction in urban pedestrian movement. Environ. Plan. B Plan. Des..

[18] Dobesova Z., Mastorakis N., Demiralp M., Baykara N.A. (2011). AICT'11: Proceedings of the 2nd International Conference on APPLIED Informatics and Computing Theory.

